# Dynamic reconfiguration of aperiodic brain activity supports cognitive functioning in epilepsy: A neural fingerprint identification

**DOI:** 10.1016/j.isci.2024.111497

**Published:** 2024-11-28

**Authors:** Emahnuel Troisi Lopez, Marie-Constance Corsi, Alberto Danieli, Lisa Antoniazzi, Marianna Angiolelli, Paolo Bonanni, Pierpaolo Sorrentino, Gian Marco Duma

**Affiliations:** 1 Institute of Applied Sciences and Intelligent Systems, National Research Council, 80078 Pozzuoli, Italy; 2 Sorbonne Université, Institut Du Cerveau – Paris Brain Institute -ICM, CNRS, Inria, Inserm, AP-HP, Hopital de La Pitié Salpêtrière, 75013 Paris, France; 3 IRCCS E. Medea Scientific Institute, Epilepsy Unit, 31015 Conegliano (TV), Italy; 4 Unit of Nonlinear Physics and Mathematical Models, Department of Engineering, Campus Bio-Medico University of Rome, 00128 Rome, Italy; 5 Institut de Neurosciences des Systèmes, Aix-Marseille Université, 13005 Marseille, France; 6 University of Sassari, Department of Biomedical Sciences, Viale San Pietro, 07100 Sassari, Italy

**Keywords:** Neurology

## Abstract

Temporal lobe epilepsy (TLE) is characterized by alterations of brain dynamic on a large-scale associated with altered cognitive functioning. Here, we aimed at analyzing dynamic reconfiguration of brain activity, using the neural fingerprint approach, to delineate subject-specific characteristics and their cognitive correlates in TLE. We collected 10 min of resting-state scalp-electroencephalography (EEG, 128 channels), free from epileptiform activity, from 68 TLE patients and 34 controls. The functional network was defined by the spatiotemporal spreading, across cortical regions, of aperiodic bursts of signals’ amplitude (neuronal avalanches), encapsulated into the avalanche transition matrix (ATM). The fingerprint analysis of the ATMs revealed more stereotyped patterns in patients with respect to controls, with the greatest stereotypy in bilateral TLE. Finally, indices extracted from individual patterns of brain dynamics correlated with the memory impairment in unilateral TLE. This study helped understand how dynamic brain activity in TLE is shaped and provided patient-specific indices useful for personalized medicine.

## Highlights

• Identification of patients with temporal epilepsy using non-linear part of the EEG signal
• Neural fingerprint differentiates between unilateral vs. bilateral temporal lobe epilepsy
• Patients with temporal lobe epilepsy are characterized by more stereotyped brain dynamics
• Memory function improves in patients whose brain dynamics resemble those of controls

## Graphical abstract

**Figure:**
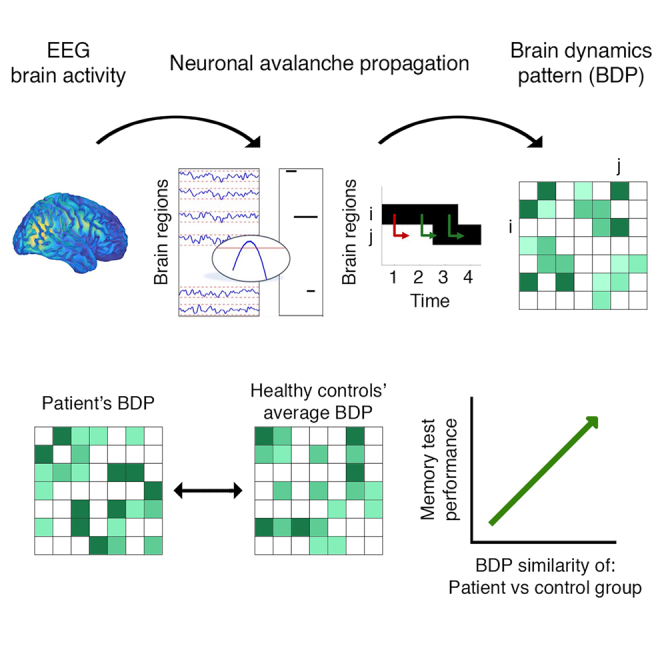

## Introduction

In the last decades, the conceptualization of epilepsy as a network disease has proved successful in improving the understanding of its pathophysiology.^1^^,^^2^ In particular, alterations of brain dynamics on the large-scale have been identified in several epilepsy types, and they have been related both to clinical and neuropsychological outcomes.^3^^,^^4^ Amongst the epilepsy types, temporal lobe epilepsy (TLE) is the most frequent drug-resistant focal epilepsy. A proportion of patients with TLE displays bilateral (simultaneous and/or independent) temporal ictal involvement, a condition defined as bilateral temporal lobe epilepsy (BTLE).^5^ Some studies have described distinctive clinical-anatomo-electrophysiological features of BTLE as compared to unilateral TLE (UTLE), suggesting that BTLE may be considered a relatively specific condition within the TLE spectrum.^6^^,^^7^ From a neurocognitive perspective, TLE has been associated with impairment in different cognitive domains, including memory, language, attention, and executive functions.^8^^,^^9^ Moreover, recent findings suggested that patients with BTLE are characterized by worse neuropsychological outcome as compared to UTLE.^10^ Converging evidence has shown a link between the disruption of large-scale functional organization in TLE and the alterations of cognitive performance in this population.^11^^,^^12^^,^^13^

Cognitive functions rely on the coordinated interactions among multiple brain areas over time. In fact, in the healthy brain, the capability of reorganization of large-scale functional properties^14^ has been related to cognitive proficiency.^15^^,^^16^^,^^17^ Relevantly, the dynamic reconfiguration of brain functional architecture over time contains enough information to unambiguously identify individuals, representing a subject-specific neural fingerprint.^18^ Alteration of the brain functional organization related to neurological diseases can induce a loss of the neural fingerprint, which has been deployed as a clinical biomarker.^19^^,^^20^^,^^21^ Clinically relevant information regarding communicative structure across brain regions can be investigated non-invasively using scalp-electroencephalography (EEG), as it represents one of the election tool in epilepsy diagnosis.^22^ Recent findings proposed an EEG derived measure, namely the microstates as a potential neural signature derived from whole-brain dynamics, differentiating not only patients from controls, but also UTLE from BTLE.^23^ Indeed, microstates are stable configurations of topographical EEG maps related to an underlying functional organization on the large scale.^24^ Despite being a promising neural marker, the investigation of functional configuration can represent a clinically applicable measure in relation to its capability of accounting for the inter-individual variability across patients.

In this perspective, the present study aims at exploiting dynamic features of brain functional architecture captured by the EEG signals in order to define the neural fingerprint of patients with epilepsy as compared to controls. The fingerprint approach represents a promising methodological approach to capture subject-wise specificity in the reconfiguration of brain dynamics. Indeed, this approach was used to measure the stability of brain patterns within a single subject (self-similarity - Iself) as well as the similarity of an individual to others within the same group (similarity with others - Iothers).^19^^,^^20^^,^^21^ This methodological framework allowed for the assessment of brain pattern stability and its characterization at both the group and individual levels, in both healthy individuals and patients with neurological and neurodegenerative disorders. This leads to the second aim of our work, which is to identify potential biomarkers that can explain specific characteristics of epileptic patients, including their particular condition (i.e., BTLE and UTLE).

To this purpose, we focused on the topography of the spreading of aperiodic perturbations across the whole-brain,^25^^,^^26^ namely the neuronal avalanches (NAs). The NAs represent the aperiodic bursts of brain activities spreading over the large scale, and are part of the critical brain hypothesis that sees the brain as operating near a critical point allowing for optimal information processing and adaptability.^27^^,^^28^ This approach allows capturing brain activity on the millisecond scale at the level of individual brain regions, and converging evidence has shown that functional connectivity is driven by these aperiodic bursty components,^29^^,^^30^ whose propagation across brain regions can be stored in matrices, that were named avalanche transition matrices (ATMs).^28^ Previous studies within the criticality framework show that critical dynamics is altered in patients with epilepsy and was linked to the epileptic activity.^31^^,^^32^ The ATMs are a TLE-sensitive measure that provide information about functionally altered regions as well as the relationship of the disrupted brain dynamics and the morphological configuration of the gray matter in patients with epilepsy.^33^^,^^34^ Moreover, the ATMs are optimally suited to capture fingerprinting, as compared to classical functional connectivity measures.^35^^,^^36^ Here, we exploited ATMs to capture the neural fingerprint of patients with TLE vs. a control group. To this purpose, we recorded 10 min of resting state activity with high-density scalp EEG (hdEEG, 128 channels) from which we performed electrical source imaging. No seizures were recorded during the resting state. However, we purposely excluded interictal epileptic discharges (IEDs) to investigate if the basal configuration of the brain, irrespective of epileptiform activities, could provide enough information to differentiate between individuals with epileptic conditions (UTLE vs. BTLE) and healthy controls.

We hypothesized that the patients may express increased differentiability as compared to controls, given the alteration observed in their dynamic activity in previous studies.^4^^,^^11^^,^^33^ In other words, the presence of the alterations in TLE provokes changes in the dynamics, such that “healthy”, optimally flexible dynamics are lost, in a way that is specific to each patient. As a consequence, we expect the patients to be more heterogeneous and therefore be less similar to each other, as compared to healthy controls which we hypothesized to be characterized by a larger within-group similarity. Conversely, the impoverished and less flexible dynamics of each patient would lead to more stereotyped functional reconfiguration patterns over time, within each patient. We expect this pattern to be more pronounced in BTLE patients as compared to UTLE patients, to be able to differentiate between these two populations.^7^^,^^23^ Finally, we hypothesized that alterations in brain fingerprinting might capture suboptimal cognitive functioning in the light of the link between altered whole-brain activity and neuropsychological impairments characterizing patients with TLE. In particular, considering that dynamic functional flexibility is one of the scaffolding elements of cognitive function, we expected to detect larger similarity of the reconfiguration patterns of activity, as compared to healthy controls, in patients with a better cognitive performance.

## Results

**Figure 1:**
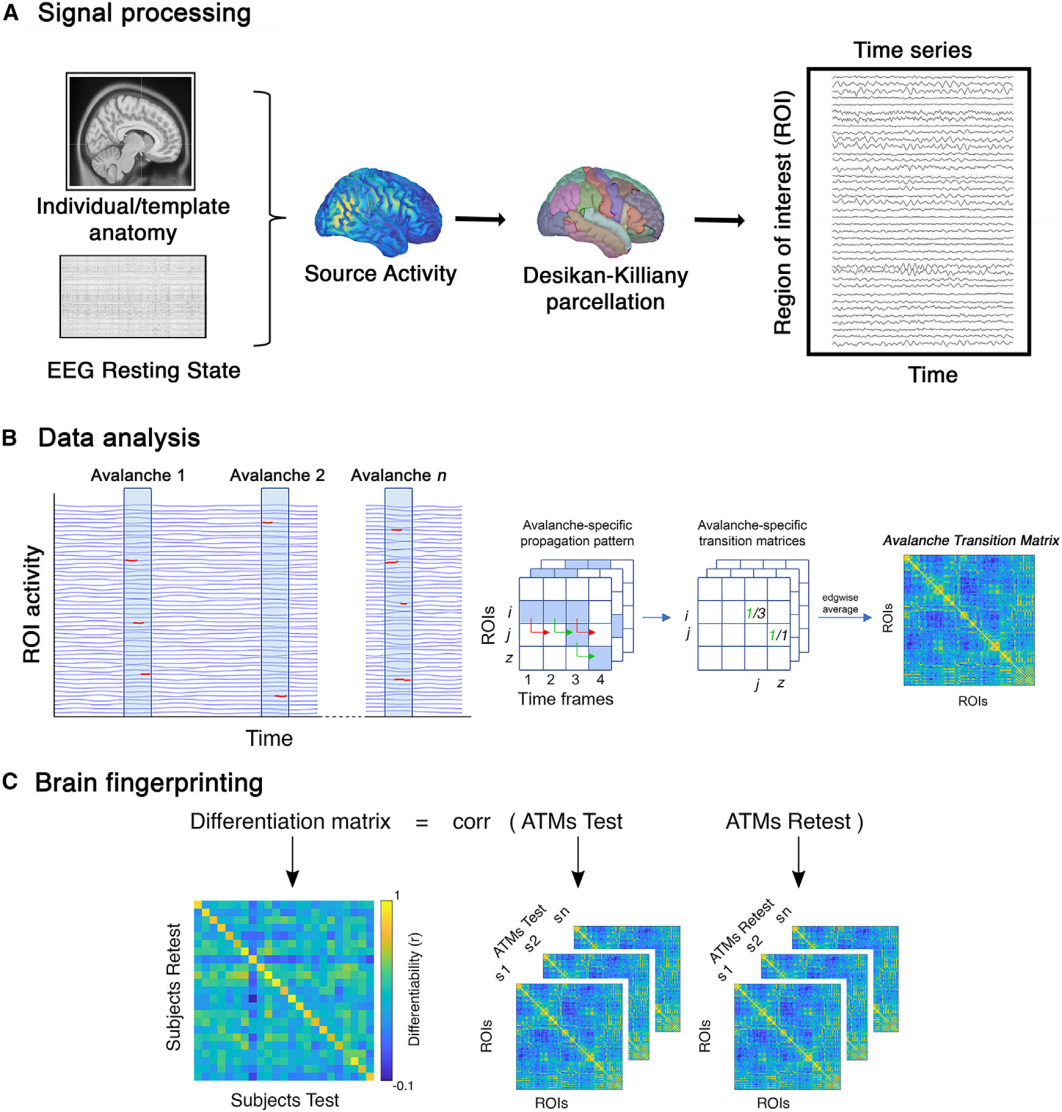
Processing and analysis pipelines (A) Displays the signal processing pipeline from resting state EEG to the source reconstruction and downsampling to a set of 68 regions of interest (ROIs) using the Desikan-Killiany cortical parcellation to extract ROIs time series. (B) Displays the data analysis pipeline: neuronal avalanches individuation; activity propagation; avalanche transition matrix (ATM).9. (C) Displays the fingerprinting analysis: test and re-test ATMs of each group were correlated, separately, obtaining a differentiation matrix for each group.

We set out to investigate brain fingerprinting in patients with TLE (see Table 1) and healthy controls, based on source reconstructed resting state scalp EEG signals (Figure 1A). Specifically, from source signals we extracted NAs (i.e., aperiodic burst of brain activity) and their pattern of propagation was stored into an adjacency matrix, the avalanche transition matrix (ATM) (Figure 1B). First, using two recordings per participant (test and retest) we correlated the ATMs within each group to build a differentiation matrix (Figure 1C). This matrix is designed to show which recordings are most similar to each other. Then, we computed three fingerprinting parameters: (1) ***Iself***: quantifying the similarity between two recordings of the same individual; (2) ***Iothers:*** the average similarity of an individual with all the others belonging to the same group; (3) ***Idiff*:** the extent to which individuals are differentiable within their group.

**Table 1:** Demographic and clinical characteristics of the patients with temporal lobe epilepsy (TLE)

*Patients with TLE*	*Mean ±Standard deviation*
*Age*	41.40 ± 17.11
*Age of onset*	23.55 ± 17.49
*Duration of Epilepsy (years)*	17.95 ± 18.29
*Number of Antiseizure Medications*	1.92 ± 1.09
***Antiseizure Medications***	***Number***
ACT	1
AZM	2
BRV	7
CBZ	14
CLB	8
CZP	2
ESL	15
LCM	15
LEV	11
LTG	6
OXC	7
PB	2
PER	15
VPA	12
ZNS	1
NO-ASMs	2
***MRI***	
***Mesial***	***Number***
HS	14
DNET	1
UKN	10
Amygdala enlargement	6

***Anterior (temporal pole)***	

FCD	12
Encephalocele	2
Gliosis	2

***Anterior + mesial***	

FCD+ HS	5
Developmental Venous Anomaly	1
***Negative MRI***	15

The table describes the demographic and clinical characteristics of the patients with temporal lobe epilepsy. Magnetic resonance imaging (MRI) abnormalities are reported by sublobar localization. The continuous variables are reported as mean ± SD. Antiseizure medication abbreviations: ACT, acetazolamide; AZM, acetazolamide; BRV, brivaracetam; CBZ, carbamazepine; CLB, clobazam; CZP, clonazepam; ESL, eslicarbazepine; LCM, lacosamide; LEV, levetiracetam; LTG, lamotrigine; OXC, oxcarbazepine; PB, phenobarbital; PER, perampanel; VPA, valproic acid; ZNS, zonisamide; NO-ASMs, no pharmacological treatment. Abbreviation of the identified anomalies on the MRI: FCD, focal cortical dysplasia; HS, hippocampal sclerosis; DNET, dysembryoplastic neuroepithelial tumors; UKN, unknown. Patients with MRI Unknown are those characterized by a potential MRI-lesion finding, namely a signal intensity alteration at the visual inspection from neuroradiologists, which however do not fall into specific neuroradiological categories.

### Fingerprinting analysis

**Figure 2:**
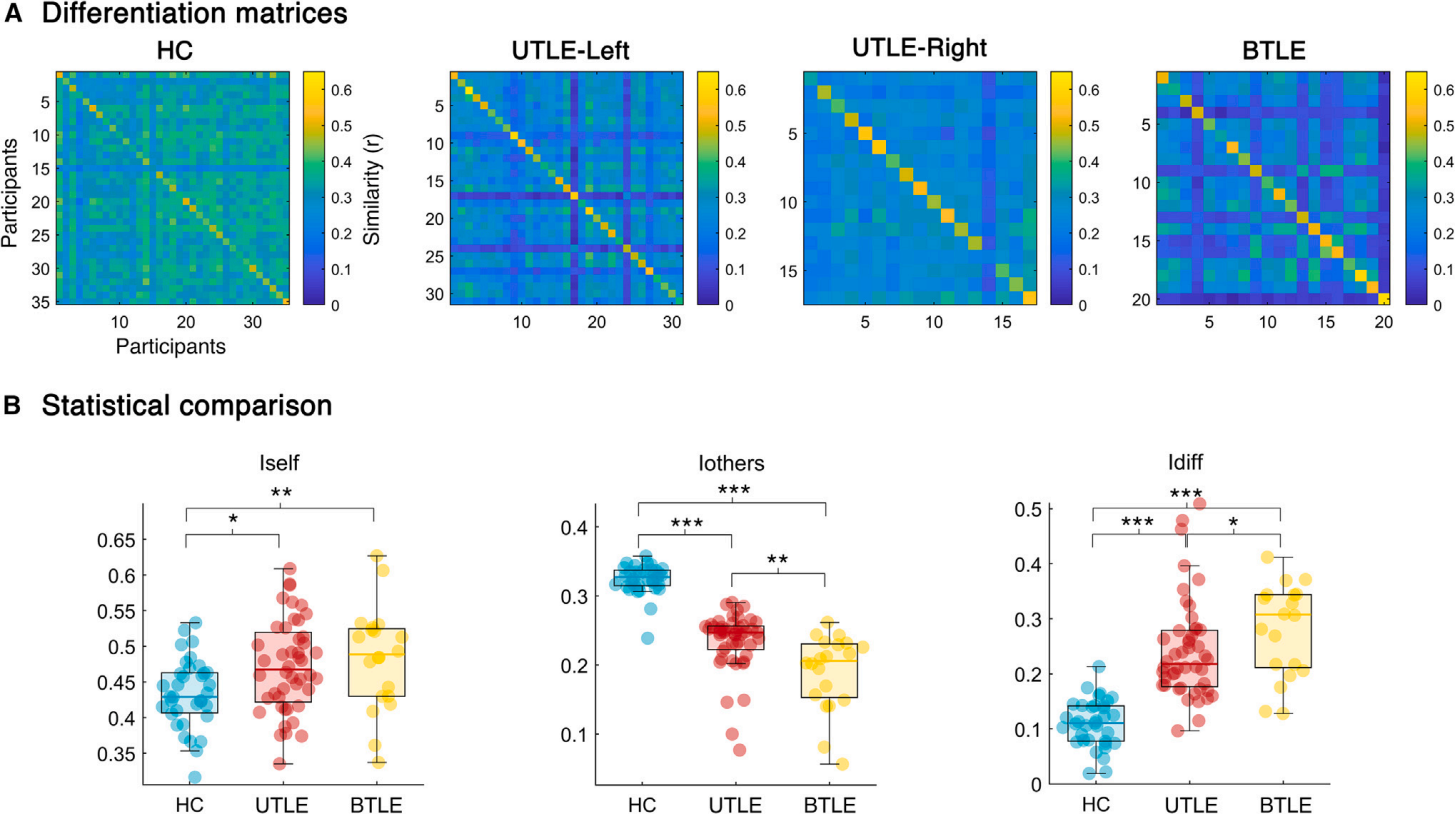
Fingerprinting analysis (A) Shows the differentiation matrices based on the avalanche transition matrix (ATM) of healthy controls (HC; *n* = 35), patients with left (*n* = 31) and right (*n* = 17) unilateral temporal lobe epilepsy (UTLE-Left and UTLE-Right, respectively) and patients with bitemporal epilepsy (BTLE; *n* = 20). The matrices present participants on rows and columns, while the elements within the matrices represent the similarity (measured using Pearson correlation coefficient (r)) between test and retest ATMs of the respective individuals. (B) Shows the statistical comparison among healthy controls, patients with unilateral temporal lobe epilepsy (both left and right) (UTLE) and patients with bilateral temporal lobe epilepsy (BTLE). Left and right UTLE patients were grouped since they did not show significant differences among themselves, while they showed the same significant differences compared to the other groups. The boxplot includes data from 25 to 75th percentiles; the median is represented by the horizontal line inside each box; error lines reach the 10th and 90th percentiles; filled circles represent the observations. Omnibus test: PERMANOVA; post-hoc analysis: permutation test; number of permutations: 10′000; *p*-values were corrected through false discovery rate (pFDR), and significance was assessed as follows: ∗ <0.05, ∗∗ <0.01, ∗∗∗ <0.001. Left panel displays Iself results (F(2,100) = 4.141, pFDR = 0.023) with post-hoc analysis (HC vs. UTLE, pFDR = 0.021; HC vs. BTLE, pFDR = 0.006). Middle panel shows the Iothers comparison (F(2,100) = 85.407, pFDR <0.001) with post-hoc analysis (HC vs. UTLE, pFDR <0.001; HC vs. BTLE, pFDR <0.001; UTLE vs. BTLE, pFDR = 0.001). Right panel shows the Idiff comparison (F(2,100) = 36.06, pFDR <0.001) with post-hoc analysis (HC vs. UTLE, pFDR <0.001; HC vs. BTLE, pFDR <0.001; UTLE vs. BTLE, pFDR = 0.048).

To investigate the differentiability of the dynamic brain pattern of TLE patients and controls, we built a differentiation matrix for each group (i.e., healthy controls, patients with left unilateral temporal lobe epilepsy [UTLE] - left, patients with right unilateral temporal lobe epilepsy - right, and patients with bilateral temporal epilepsy) (Figure 2A). Then, we compared the brain fingerprinting parameters (i.e., Iself, Iothers, Idiff) of the patients with the controls’ ones. Patients with UTLE were grouped since they did not show significant differences among themselves, while they showed the same significant differences compared to the other groups. The test performed on the ***Iself*** parameter, which provides a measure of the similarity between test and retest ATMs of the same individual, displayed significant differences among the three groups (F(2,100) = 4.141, pFDR = 0.023). In particular, we found significant lower ***Iself*** values in healthy controls with respect to patients with unilateral temporal lobe epilepsy (UTLE) (pFDR = 0.021) and bilateral temporal epilepsy (BTLE) (pFDR = 0.006). Additionally, we observed a significant effect related to the ***Iothers*** parameter (F(2,100) = 85.407, pFDR <0.001). In this case, not only healthy controls presented larger ***Iothers*** values than patients with UTLE (pFDR <0.001) and BTLE (pFDR <0.001), but we also found that the UTLE displayed higher ***Iothers*** values than BTLE (pFDR = 0.001). Finally, we also found a significant effect of the ***Idiff*** (F(2,100) = 36.06, pFDR <0.001). In particular, we found lower ***Idiff*** values in healthy controls compared to UTLE (pFDR <0.001) and BTLE (pFDR <0.001). Relevantly, patients with BTLE were characterized by larger ***Idiff*** values as compared to UTLE (pFDR = 0.048). The statistical analysis was repeated using recording trials of 180 s and is available in the supplemental information document (Figure S1).

### Stability of brain activity

**Figure 3:**
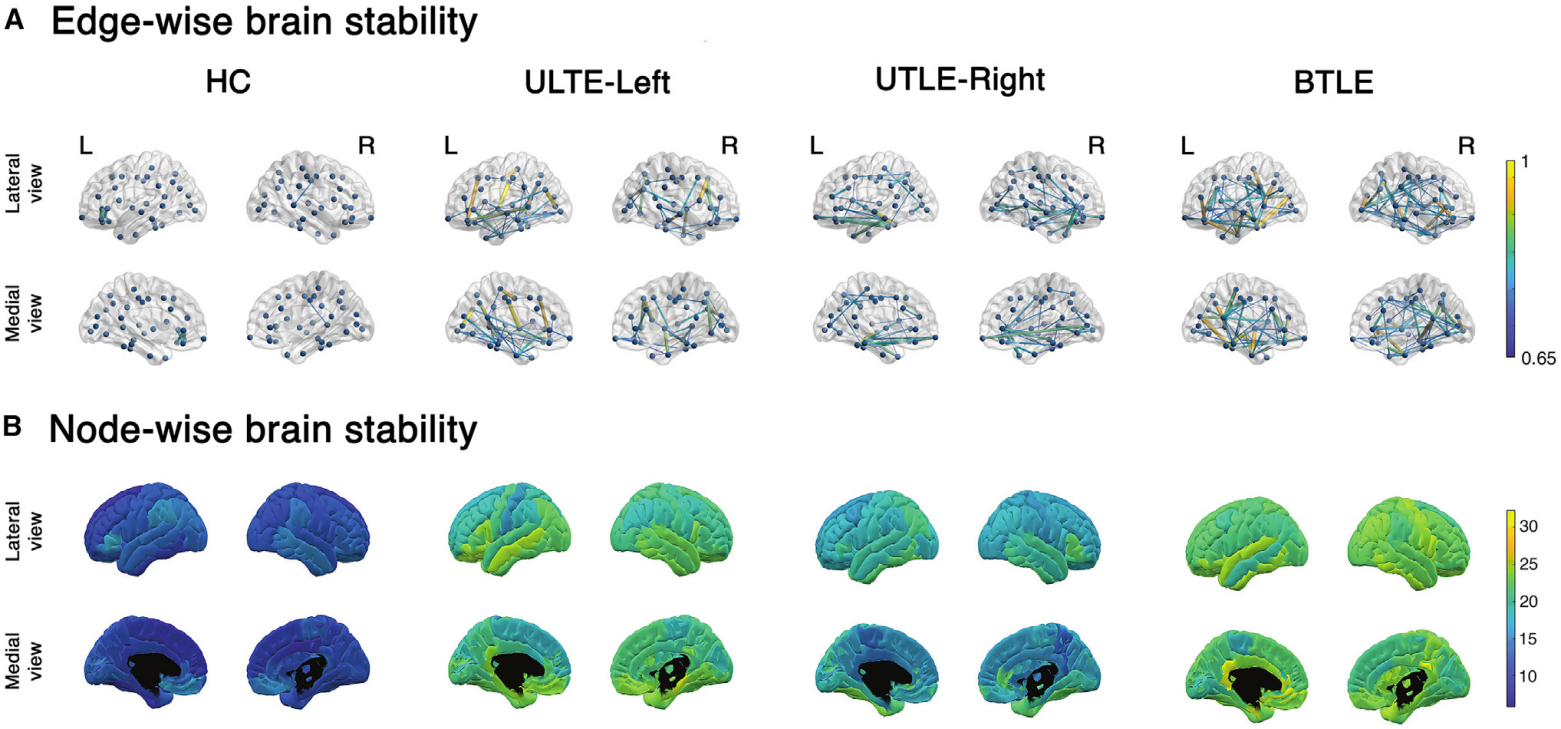
Stability of brain activity Brain plots revealing the test-retest stability of nodes and edges in each group, obtained by the means of intraclass correlation analysis. Healthy controls (HC; *n* = 35), patients with left (UTLE-Left; *n* = 31) and right (UTLE-Right; *n* = 17) unilateral temporal lobe epilepsy, and patients with bilateral temporal lobe epilepsy (BTLE; *n* = 20). (A) The figure shows the edge-wise stability from lateral and medial views of both brain hemispheres; dots represent the brain regions; bars connecting the dots represent the stability of the link between two given nodes (for visualization purposes, only edges when ICC ≥0.65 are displayed). The higher the stability, the thicker the link. (B) The figure shows the nodal stability from lateral and medial views.

We successively investigated the stability of the functional links across regions, namely the edges of the ATMs, according to each group of participants. Stability was assessed by the means of intraclass correlation coefficient (ICC). Specifically, the higher an ICC value the higher the stability of a given edge across the test-retest recordings of the examined group. In this case, we separated the right and left UTLE participants, as this analysis considers the values of each specific edge across the participants. Figure 3 shows the regional contribution to the edges’ stability in the four groups, both edgewise (Figure 3A) and nodewise (Figures 3B and S2). Then, the average stability (mean value across all edges) was calculated for each participant and compared between groups. The HC displays the lowest stability globally (ICC mean in HC = 0.154, *p* < 0.001 vs. all patients’ groups; UTLE-Left mean = 0.339, vs. UTLE-Right mean = 0.267, vs. BTLE mean = 0.36), suggesting that there is higher heterogeneity in the spreading patterns in physiological conditions. Conversely, patients display higher stability and this alteration is mainly distinct in the bitemporal condition. It is interesting to note the marked involvement of the temporal lobe in patients with left UTLE. In the right UTLE the corresponding lobe does not display a similar behavior, while in bitemporal condition the involvement can be mainly observed in the left lobe. Details on the regions involved are displayed in Figure S3.

### Clinical correlation

**Figure 4:**
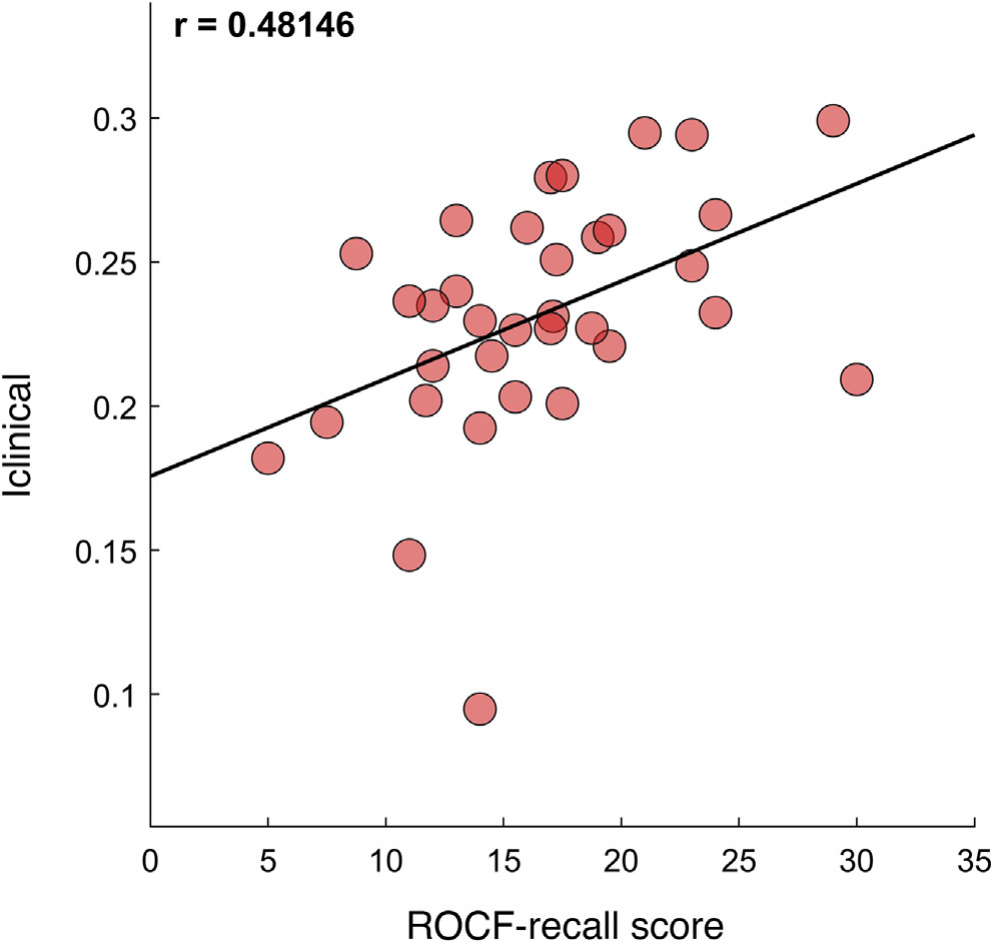
Clinical correlation The figure shows the scatterplot of the correlation between Iclinical values (i.e., similarity of the pattern of brain dynamics between a patient with the average of the control group) and recalling performance scores during the Rey–Osterrieth complex figure test (ROCF-recall), with regards to the patients with unilateral temporal lobe epilepsy. The more the patients were similar to the healthy controls (higher Iclinical), the better the recalling performance. Red dots represent the observations. Statistical test: Pearson correlation test (r = 0.481, *p* = 0.004); *n* = 34 out of 48 total patients with unilateral temporal lobe epilepsy, as clinical data were not available for 14 patients.

Borrowing from previous studies we calculated the ***Iclinical*** score, which represents how much the ATM of a patient resembles the average ATMs of the healthy controls. We found that, in patients with UTLE, the ***Iclinical*** was significantly correlated to the score of the figure recall test (ROCF-recall) (r = 0.48, pFDR = 0.032) (Figure 4). The correlation was performed on 34 out of 48 patients, because neuropsychological scores were not available for 14 patients. None of the remaining neuropsychological variables (Table 2) displayed significant correlations. The correlation test was repeated using recording trials of 180 s and is available in the supplemental information document (Figure S4). Additionally, we tested for possible correlations between all fingerprint parameters and the number of medications taken by the patients, but found none (Table S1, supplemental information). This, along with the fact that 90% of the population (61 out of 68) was resistant to the medications, reduces the risk that the results could be driven by medication administration.

### Multilinear regression analysis for clinical prediction

**Figure 5:**
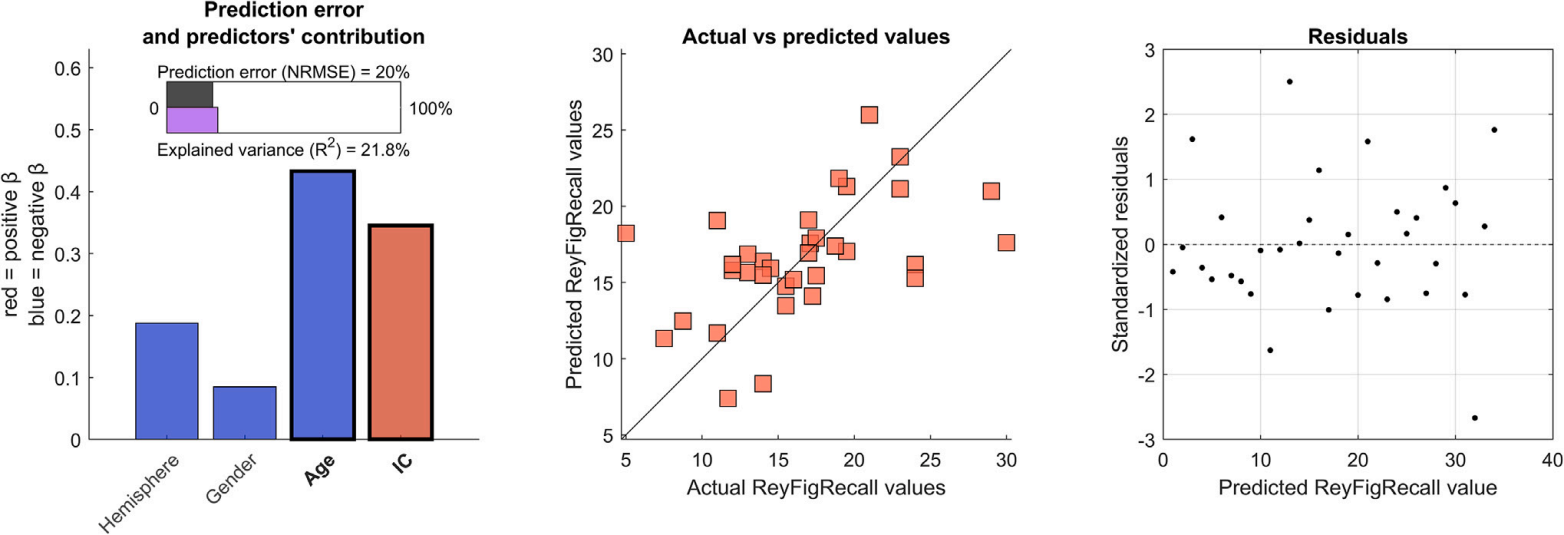
Multilinear regression model for clinical prediction The figure shows the results of the multilinear model with nested 5-fold cross-validation. The multilinear model significantly predicts the scores of the Rey–Osterrieth complex figure recall test (ROCF-recall) in patients with unilateral epilepsy (F(4,29) = 4.39, *p* = 0.007). The model is based on four predictors (i.e., lateralization of the condition (Hemisphere, in figure), Gender, Age, and Iclinical (IC)). The left panels report the statistics of the model; predictors’ values are z-scored in order to make the beta coefficients comparable; significant predictors are reported in bold (Age, β = −0.43, *p* = 0.016; IC, β = 0.35, *p* = 0.0489); NRMSE: normalized root mean square error is equal to 20%; explained variance is represented by R2 measure, equal to 21.8%. The middle panel is a scatterplot that compares the actual ROCF-recall scores with the ROCF-recall scores predicted by the model. The more the predictors are aligned along the diagonal, the higher is the accuracy of the prediction; Spearman correlation coefficient = 0.718. Finally, the third panel shows the distribution of the standardized residuals. Sample size is *n* = 34, out of 48 total patients with unilateral temporal lobe epilepsy, as clinical data were not available for 14 patients.

Furthermore, we also tested the ability of the ***Iclinical*** score to predict, together with other predictors (i.e., affected hemisphere, gender, and age), the ROCF-recall scores. Hence, we built a multilinear regression model validated with a nested 5-fold cross validation (Figure 5) over 4000 iterations, and found that both age (β = −0.43, *p* = 0.016) and Iclinical (β = 0.35, *p* = 0.0489). The cross-validated model resulted to be significant (F(4,29) = 4.39, *p* = 0.007), with an explained variance equal to 21.8% (R2 = 0.218), a prediction error equal to 20% (NRMSE = 0.2), and a Spearman correlation coefficient between predicted and actual ROCF-recall scores equal to 0.718. The multilinear regression model was also tested based on the ***Iclinical*** scores calculated from 180-s recording trials and is available in the supplemental information document (Figure S5).

## Discussion

In this study, we set out to investigate whether brain dynamics may represent a neural fingerprint to identify individuals and their clinical condition, namely epilepsy. We leveraged previous findings showing that NAs represent a sensitive measure able to capture the altered functional organization in epilepsy.^33^^,^^34^ Recent findings highlighted that ATM increase the performance in subject identification, i.e., neural fingerprint.^36^ In this light, we quantified the similarity between the ATMs across and within groups, to test the hypothesis that the changes in large-scale dynamics may characterize the individual neural fingerprint and differentiate between patients and controls. While multiple studies have demonstrated altered functional configurations in TLE, we chose to focus on changes in individual patients as compared to the healthy controls to better incorporate the intra-individual variability characterizing this pathology.

As a first result, we observed that patients diverge from the ‘healthy’ optimal configuration observed in controls, as they display more stereotyped dynamics. As such, each patient is more similar to him/herself over time (larger ***Iself*** values) and less similar to the other patients (reduced ***Iothers*** value). Then, we chose to analyze in depth what edges were driving the differences in identifiability. The edge-based results provide an additional piece of information on the global dynamics of patients. Healthy controls were characterized by more flexible brain dynamics configurations, as shown by the lower number of stable connections across brain regions, as compared to patients. Conversely, TLE patients showed more stable edges, and more stereotyped dynamics of the frontotemporal regions. Importantly, the number of stable edges increased from unilateral to bilateral TLE (see Figure 3).

At first, these findings highlight that epilepsy is a network disorder impacting the brain dynamics at the whole-brain level. Second, while TLE may be considered a homogeneous clinical category, there is an array of clinical presentations according to the portion of the lobe involved in seizure generation.^37^^,^^38^^,^^39^ Such heterogeneity may be mirrored in corresponding variability of brain dynamics on the large scale. Our findings, while corroborating the concept of TLE as a heterogeneous category, provide additional insight into the possibility of individual identification based on the functional reconfiguration of the brain networks, leveraging the concept of personalized medicine. Additionally, the brain dynamics organization represents a sensitive measure of the lateralization of the clinical condition. In fact, we observed decreased ***Iothers*** value in the BTLE patients, supporting a difference in the functional configuration of brain activity as compared to the UTLE. Accordingly, BTLE has been proposed as a separable and specific condition as compared to UTLE.^7^^,^^40^ Recent evidence suggests increased segregation and lower global efficiency in the functional networks of patients with BTLE.^41^ Our results align with the observed altered segregation/integration ratio in BTLE, highlighting a reduction in the repertoire of brain activity reconfiguration, resulting in more stereotyped dynamics in this population.

A high degree of intra-individual variability in brain activity patterns can be interpreted as an indicator of a healthy brain. This concept is based on the idea that the variability reflects the ability of the brain to flexibly adapt to multiple cognitive and behavioral tasks. To achieve this ability, the brain alternates moments of coherent activities over the large scale (integration) with moments of rearrangement of the activities (segregation) where no obvious pattern is observed on the large scale. The fine-tuning of the integration-segregation ratio^42^ is considered to be optimizing the system capability to efficiently process environmental stimuli, while minimizing potential damage.^43^ The physiological variation in brain activity patterns reflects the complexity and uniqueness of each individual, configuring a “neural fingerprint”. However, it must be noted that quantifying the trade-off between variability/flexibility is challenging, and it can only be achieved as relative to the (presumably) optimal configuration observed in the healthy controls. In this case, brain pathology is often associated with a loss of flexibility and the emergence of stereotyped activities.^44^ In neurological and psychiatric disorders, rigid and repetitive brain activity patterns are often observed, which has been related to cognitive impairment.^45^^,^^46^ Importantly, patients with TLE are characterized by the impairment of multiple cognitive domains, which has been linked to the dysregulation of reconfiguration properties of brain dynamics at the large scale.^12^^,^^13^^,^^47^ Our results showed that the more the patterns of propagation of the whole-brain dynamics in patients resembled those of healthy controls, the better the cognitive proficiency, in this limited to the long-term memory (recall of the ROCFT).

Overall, our results corroborate the use of ATMs as a straightforward way to capture subject-specific, large-scale spatiotemporal dynamics. While the alteration of brain dynamics in epilepsy has already been shown,^11^^,^^13^^,^^48^ the approach proposed here provides translational potential toward clinical applications as well as an advancement of the understanding of the large-scale neurophysiological mechanisms characterizing epilepsy. In fact, the majority of the previous studies leveraging the functional connectome in epilepsy were related to group-based measures, while neural fingerprint accounts for the inter-individual variability, generating indices which can be used in a patient-specific fashion. Furthermore, the ***Iclinical*** score represents an index measuring the similarity of patients with respect to a reference population, which might be suitable for clinical practice. Moreover, the network behavior description, here adopted, is related to the theoretical formalization of brain criticality which has been proved to be useful in describing the activity of neural assemblies at multiple scales, with implication in the generation of critical and intercritical epileptiform activity.^49^^,^^50^ Relevantly, higher order interactions (i.e., derived by non-linear process), namely those occurring during NAs, are required to account for cortical dynamics.^51^ The ATM has proved to be a useful tool to quantify such higher-order large-scale dynamics and therefore to better characterize neural activity both in healthy and clinical populations as compared to classic phase/power based connectivity metrics.^28^^,^^36^^,^^52^

Our investigations were indeed limited to the cortical dynamics. This limitation is related to the relatively low spatial resolution of the EEG for subcortical areas.^53^^,^^54^ Future investigations could also consider the study of the contribution of subcortical areas. The thalamus represents indeed a specific target for future studies considering recent findings emphasizing its contribution in the specialization of functional organization,^55^ and its mediating role in the propagation of temporal seizures and the promotion of cortical synchronization.^56^ Despite the spatial limitation for the investigation of subcortical dynamics, the EEG represents one of the election tools in the diagnosis of epilepsy. The cost-effective and portable nature of this instrument makes EEG-derived neural markers of important potential clinical applicability. In the present work the ATMs have proved to be an EEG-derived measure sensitive to pathology-induced alterations of brain dynamics, being able to discriminate between controls and patients with TLE. While spatial-specificity might be suboptimal, given the use of a default anatomy for the control group, the differences of the functional organization across groups remain a stable and reliable finding. Additionally, by using ATMs we have been able to provide insights on the neurofunctional mechanisms distinguishing UTLE from BTLE. Moreover, our findings endow the reconfigurations of neural activity with a functional meaning since they relate to a cognitive process that has been long known to be impaired in patients with TLE, namely memory. Importantly, our results are based on a signal cleaned from epileptiform activity with 2-fold implications. At first from a theoretical perspective we highlighted how the basal process of regulation of neural dynamics is altered in this clinical condition. Second, having a pathology-sensitive metric, characterizing individual patterns of neural activity without the need of epileptiform activities, drastically increases the usability in a real clinical scenario. Finally, providing substantially unchanged results using both 100-s trials and 180-s trials, we can assert that our findings do not depend on the amount of time analyzed, at least within the considered range of time. This opens new possibilities for a tailored investigation of brain dynamics in disease conditions, providing metrics for a fine-tuning of patient-specific brain models.

### Limitations of the study

This study aimed at investigating the subject-specific patterns of brain dynamic activity in TLE patients, to highlight specific markers related to clinical aspects. One limitation of this study concerns the small sample size of the group of patients with right UTLE. Future studies that include an equivalent number of patients with both right and left unilateral TLE may allow for a more thorough investigation of any unique characterizations of the specific subtype. Similarly, it would be beneficial for future studies to include different forms of epilepsy to determine the potential specificity of our results for TLE. The study sample was composed by white and Italian participants, therefore future studies should include different ethnicities to better account for generalizability of the results across different populations. Additionally, our control displayed slight age differences as compared to the patients. The investigated age intervals both for control and the epilepsy group are characterized by a full-matured brain in the adult stage. Nonetheless, future studies may investigate how neural fingerprint parameters change across ages, with a life-span approach. However, the brain fingerprinting approach has enabled us to extract highly consistent subject-specific data within specific patient subgroups, providing solid findings.

## Resource availability

### Lead contact

Gian Marco Duma (gianmarco.duma@lanostrafamiglia.it).

### Material availability

The data that support the findings of this study are available on request to the corresponding author. The raw data are not publicly available due to privacy or ethical restrictions.

### Data and code availability

• The raw data are not publicly available due to privacy or ethical restrictions.
• The main codes utilized for the analysis are available at the following links: https://github.com/pierpaolosorrentino/Transition-Matrices-.git, https://github.com/eamico/Clinical_fingerprinting.git.
• Any additional information required to reanalyze the data reported in this work paper is available from the lead contact upon request.

## Acknowledgments

European Union “10.13039/100031478NextGenerationEU”, (Investimento 3.1.M4. C2), project IR0000011, EBRAINS-Italy of PNRR. Funds for biomedical research of the 10.13039/501100003196Italian Ministry of Health: Ricerca Corrente 2024.

## Author contributions

Conceptualization: E.T.L. and M.C.C.; Methodology: E.T.L. and M.C.C.; Software: E.T.L. and M.A.; Formal analysis: M.C.C.; Investigation: A.D. and P.B.; Data curation: L.A. and G.M.D.; Resources: L.A.; Visualization: M.A.; Project administration: P.S. and G.M.D.; Supervision: P.S. and G.M.D.; Funding acquisition: E.T.L. and G.M.D.; Writing – original draft: E.T.L., M.C.C., P.S., and G.M.D.; Writing – reviewing and editing: E.T.L., M.C.C., A.D., L.A., M.A., P.B., P.S., and G.M.D.

## Declaration of interests

The authors declare no competing interests.

## STAR★methods

### Key resources table

**Table:** 

REAGENT or RESOURCE	SOURCE	IDENTIFIER
**Software and algorithms**		

Code used for the analysis	https://github.com/pierpaolosorrentino/Transition-Matrices-.git https://github.com/eamico/Clinical_fingerprinting.git	N/A
**EEGLAB**	https://github.com/sccn/eeglab	Article: https://doi.org/10.1016/j.jneumeth.2003.10.009
**Brainstorm**	https://github.com/brainstorm-tools/brainstorm3	Article: http://www.hindawi.com/journals/cin/2011/879716/

### Experimental model and study participant details

The research was conducted in accordance with the Declaration of Helsinki. A written informed consent was obtained from subjects after explanation of the study, which was approved by the ethical committee “Comitato Etico Area Nord Veneto” (number protocol: 0001878/24).

We retrospectively enrolled 72 patients with temporal lobe epilepsy, who underwent scalp high-density electroencephalography (hdEEG, 128 channels) for clinical evaluation between 2018 and 2022 at the Epilepsy and Clinical Neurophysiology Unit, IRCCS Eugenio Medea in Conegliano (Italy). The diagnostic workflow included clinical history and examination, neuropsychological assessment, long-term surface Video EEG (32 channels) monitoring, high-density EEG (hdEEG) resting-state recording, magnetic resonance imaging (MRI) of the brain, and positron emission tomography (PET) as an adjunctive investigation in selected cases. The diagnosis of temporal lobe epilepsy was established according to the ILAE guidelines. A number of 4 patients received invasive surgery before the hdEEG recording. For this reason, we reduced the final sample size to 68 (31 left-TLE; 17 right-TLE; 20 bilateral TLE) (whole sample mean age = 41.40 [SD = 17.11]; 33 females). A description of patients’ demographic and clinical characteristics is provided in Table 1, while the details based on the specific subgroup of patients are reported in Table S2 and Table S3. The control group sample size was composed of 35 healthy participants with no history of neurological or psychiatric disorders (mean age = 34.92 [SD = 9.22]; 25 females). The participants in the study are all White and Italian. The ancestry of the participants is not known as this information was not included in the protocol approved by the ethics committee. Information related to species/strain, genotype, age/developmental stage, sex, maintenance, and care, including institutional permission and oversight information for the studies the experimental animal/human participant study are mentioned above and reported in Table S3. The influence (or association) of sex, gender, or both on the results of the study are reported in the Results details (see Figure 5).

**Table 2:** Neuropsychological scores

Test	Score (mean ± standard dev)
***DigitSpan***	5.63 ± 1.12
***Corsi block Tapping Test***	4.83 ± 1.05
***ROCFT - Copy***	32.06 ± 4.83
***ROCFT - Reproduction***	15.11 ± 6.48
***RAVLT - Immediate***	39.12 ± 9.02
***RAVLT - Delayed***	7.20 ± 3.23
***TMT-A***	33.01 ± 16.30
***TMT-B***	113.48 ± 72.91

The present table shows the mean value across groups of the neuropsychological performance. Abbreviation: RAVLT = Rey auditory verbal learning test; ROCF = Rey–Osterrieth complex figure test; TMT A/B = trail making test A/B.

### Method details

#### Resting state EEG recording

The hdEEG recordings were obtained using a 128-channel Micromed system referenced to the vertex. Data was sampled at 1,024 Hz and the impedance was kept below 5kΩ for each sensor. For each participant, we recorded 10 min of closed-eyes resting state while comfortably sitting on a chair in a silent room.

#### EEG pre-processing

EEG signals were preprocessed offline via EEGLAB 14.1.2b 22.^57^ The first step consisted of a downsampling at 250 Hz followed by a [0.1–45Hz] bandpass-filtering with a Hamming windowed sinc finite impulse response filter (filter order = 8250). Interictal epileptiform discharges (IEDs) were identified via a visual inspection made by the clinicians. The signals were cut into 1-s epochs. To focus on the intrinsic brain functional organization independently from epileptiform activities, epochs that contained IEDs were removed. Then, the detection of bad channels and of the artifacts was performed automatically via the TBT plugin implemented in EEGLAB. The associated algorithm consisted of the identification of the channels that exceed a differential average amplitude of 250 μV and labeled them as “bad channels”. Channels that were labeled as such in more than 30% of all the epochs were removed. Besides, epochs with more than 10 channels labeled as “bad” were also excluded. Flat channels were detected via the Trimoutlier EEGLAB plug-in by applying a lower threshold of 1 μV. On average, we rejected 55.04 ± 52.96 (SD) epochs related to IED and artifacts. Such a preprocessing pipeline has been applied by our group in previous studies that investigated neuronal avalanches in epilepsy with resting state EEG activity.^33^^,^^52^ Artifact removal was performed via independent component analysis, with the Infomax algorithm implemented in EEGLAB. After a visual inspection of the 40 independent components (ICs), the ICs that contained eye blinks, eye saccades, muscular or cardiac artifacts were removed. The preprocessed signals consisted of the projection of the remaining ICs remaining components into the electrode space. The data were re-referenced to the average of all electrodes. The resulting data consisted of at least 6 min of artifact-free signals for each subject.

#### Cortical source modeling

To generate individual head models for the patients with TLE, we used the individual MRI anatomy that consisted of a T1 isotropic three-dimensional (3D) acquisition. MNI-ICBM152 default anatomy^58^ from Brainstorm^59^ was used for 12 patients and for the control group since the 3D T1 MRI sequences were not available. To segment the MRI into skin, skull, and gray matter, we used the Computational Anatomy Toolbox (CAT12).^60^

Individual surfaces were then imported in Brainstorm to apply the Boundary Element Models (BEM) method to reconstruct three surfaces (namely the inner skull, the outer skull, and the head). The cortical mesh was downsampled at 15,0002 vertices. To co-register the EEG electrodes via Brainstorm, we projected the EEG sensor positions onto the head surface by using the fiducial points of the individual or the template MRI (Figure 1A). Before projecting the electrodes on the individual head surface, whenever needed, we applied a manual correction of the EEG cap on the individual anatomy. The EEG forward model was derived using the 3-shell BEM model (conductivity: 0.33, 0.165, 0.33 S/m; ratio: 1/20) estimated via OpenMEEG method implemented in Brainstorm.^61^^,^^62^

Finally, we estimated the sources with the weighted minimum norm estimate^63^ with the Brainstorm’s default parameter setting.

#### Brain dynamics

To explore the dynamics of brain activity, we derived "neuronal avalanches" from the time series reconstructed at its source (Figure 1B). Firstly, for a fair comparison we used the same duration for each recording of each participant. For a fingerprint analysis, we needed two recordings (test and retest) for each individual hence we split the recordings in two segments of equal duration (i.e., 180 s per trial). Then, we selected 100 s from each trial, starting from a random point in time, to avoid biases such as consistently taking the initial part of all recordings. However, the analysis was repeated using the maximum length of data available (i.e., 180 s) and all the results were confirmed and reported in the supplemental information document (see Table S4). In both cases, the subsequent step consisted in discretizing the time series for each region of interest by calculating the *Z* score as follows:

$$Z\left(t\right)=\frac{\left(x_{t}-\mu \right)}{\sigma }$$

where x is the signal, μ is the average value of the signal across time, and σ is its standard deviation.

Subsequently, we detected both positive and negative excursions surpassing a specified threshold *Thres*, as:

X(i,t)={1ifabs(X(i,t)>Thresh,0otherwise}

where, *X* is the matrix of time series, with *i* representing the regions, and *t* representing the time. With this procedure, the matrix of time series became logical, reporting 0 when the amplitude of the signal was under threshold, and 1 when the amplitude was above threshold (i.e., burst of activation). Specifically, the analyses were performed setting the threshold at 2.8 standard deviations, and then repeated at 2.6 and 3 standard deviations, to check that the results were not dependent on a single specific threshold. A neuronal avalanche begins when, in a sequence of contiguous time bins, at least one ROI is active (i.e., the amplitude of the signal is above threshold), and ends when all ROIs are inactive.^28^ Given the sampling rate of our data (i.e., 256Hz), each time bin contained 4 ms of recording. Then, to ensure that we were observing a system operating in a near-critical regime, we calculated the branching ratio (i.e., a measure that characterizes the division or divergence of pathways within a structure or process), that in systems operating at criticality typically displays value ∼1.^64^ Specifically, the branching ratio was determined by geometrically averaging the ratio of the number of events (activations) between subsequent time bins and the current time bin, over all time bins, and then averaging it across all avalanches, as:

$$\sigma_{i}=\prod_{j=1}^{N_{bin}-1}{\left(\frac{n_{events}\;\left(j+1\right)}{n_{events}\;\left(j\right)}\right)}^{\frac{1}{N_{bin}-1}}$$

$$\sigma =\prod_{i=1}^{N_{aval}}{\left(\sigma_{i}\right)}^{\frac{1}{N_{aval}}}\;$$

Where σi is the branching parameter of the i-th avalanche in the subject, Nbin is the total amount of bins in the i-th avalanche, Naval is the total number of avalanches in the dataset. σ is the branching ratio of the referred to the whole recording. Hence, we calculated the σ value of the unbinned time series, and then the σ values when applying the binning at 2 and 3 time bins (8 and 12 ms, respectively).^65^ Then we continued the analysis using the time series that showed the σ value closest to 1, which was represented by the unbinned time series.

Then, for each avalanche n, the transition matrix AvalATM (n) was defined as:

$$AvalATM\left(i,j\right)=P\left(X_{\left(j,t+ẟ\right)}>Thres\;|\;X_{\left(i,t\right)}>\;Thres\right)$$

where the element (i, j) represents the probability that region j is active at time t+ẟ, given that region i was active at time t, where ẟ∼3ms. The ATMs were averaged within each participant, as:

$$ATM\;\left(i,j\right)=\frac{1}{Naval}\sum_{n=1}^{Naval}avalATM\left(i,j,n\right)$$

and finally symmetrized. Introducing a time delay diminishes the likelihood that our findings can be easily attributed to field spread.^28^ Field spread refers to the simultaneous detection of multiple sources by various sensors, leading to spurious zero-lags correlations in the recorded signals.

#### Fingerprint analysis

We based our fingerprinting analysis on the brain dynamics by the means of ATMs, similarly to our previous work.^36^ Initially, our goal was to construct an Identifiability Matrix (IM) following the methodology outlined by Amico and Goñi (Figure 1C).^66^ The IM organizes participants into rows and columns, with entries representing Pearson’s correlation coefficients between the test and retest ATMs for each participant. The IM encapsulates information on self-similarity (Iself, found on the main diagonal elements), indicating the comparison of test and retest ATMs for the same participant. Additionally, it includes the similarity of each subject with others (Iothers, off-diagonal elements), signifying the resemblance between different individuals of the same group. Then, by computing the difference between the ***Iself*** and ***Iothers***, we derive the differential Identifiability (Idiff)^20^^,^^66^ that provides an estimate of the fingerprint level within a specific group. Lastly, by correlating the test-retest ATMs of healthy individuals and patients, we can obtain the Iclinical score (referred to as "clinical identifiability" or "clinical fingerprint"). This score reflects the similarity of a patient in comparison to healthy subjects. For a more in-depth understanding, please consult.^20^

#### Edges’ stability

Then, we wanted to assess the stability of pathways of activation represented by the edges of the ATMs. Building upon earlier work on identifiability,^66^ we utilized the intraclass correlation coefficient (ICC)^67^ to assess the edge-wise reliability of individual connectomes. Edges exhibit high ICC values when they consistently demonstrate similar levels of synchronization across test-retest sessions.

#### Neuropsychological assessment

All the patients (UTLE and BTLE) underwent a neuropsychological assessment focusing on memory, attention/executive functions and intelligence using the standardized tests suggested by the neuropsychological evaluation guidelines of the national (Italian) League Against Epilepsy (LICE; https://www.lice.it/pdf/Anamnesi_Neuropsicologica_LICE.pdf).

Specifically, verbal and visuo-spatial short-term memory (STM) was investigated with Digit Span Test and Corsi block tapping test, respectively.^68^ In the digit span patient is required to repeat a string of numbers which is made progressively longer in order to determine the amount of information stored in STM (i.e., amount of numbers the patient can recall). On the other hand, the Corsi block-tapping test involves mimicking the tapping sequence of identical separated blocks, performed by the clinician. The number of corrected blocks is defined as the Corsi’s number, and quantifies the amount of information stored in visual-STM.

The Rey–Osterrieth Complex Figure Test (ROCFT)^69^ was adopted to measure visual long term memory (LTM). Patients are required to copy the complex figure and then, after 5 min, reproduce it from memory. The test is useful for evaluating neurological dysfunction in visual perception and visual LTM. Successively, the Rey Auditory Verbal Learning Test (RAVLT)^70^ is performed to measure verbal LTM. The RAVLT requires to learn and to immediately recall a list of 15 words in five consecutive learning trials, free recall after distraction, as well as free recall and recognition of the target words after a 15 min delay.

Attention and executive functions were evaluated with the Trail Making Test (TMT).^71^ Specifically, the test consists of connecting 25 consecutive targets on a sheet of paper. The test consists of two versions: A and B. In TMT-A (Trail-making test A) the 25 targets are numbers (1,2,3, etc.); while in TMT-B (Trail-making test B) the targets are both numbers and letters and the subject has to alternate between them in ascending order (1, A, 2, B, etc.). The subject’s task is to connect the target stimuli with a line in the shortest possible time. Performance is measured by taking into account the time it takes the subject to complete the task. The TMT-A and TMT-B are a measure of motor speed and attention shifting capabilities, respectively. In the assessment of executive functions, the difference between TMT-B-A times is considered as indicative. The Wechsler Adult Intelligence Scale IV was used as a measure of the global IQ.^72^

Table 2 shows the descriptive statistics of the neuropsychological scores.

### Quantification and statistical analysis

The statistical analysis was conducted using MATLAB 2020a. To compare ***Iself***, ***Iothers***, and ***Idiff*** values among the three groups, a PERMANOVA test with 10,000 permutations was employed. Pairwise post-hoc comparisons were executed through permutation testing, involving the random rearrangement of labels for the two groups 10,000 times. At each iteration, the absolute value of the difference was computed, resulting in a distribution of randomly determined differences.^73^ This distribution was then compared to observed differences to determine statistical significance. The potential relationships between variables were explored using Pearson’s correlation and a multilinear regression model with nested k-fold cross-validation (k = 5), averaged over 4000 iterations, preventing overfitting and information leakage.^74^ After dividing the sample into 5 subgroups (outer loop), one of the subgroups served as the test set, while the remaining 4 subgroups were combined and entered into an inner loop, where they were again divided into 5 subgroups. In turn, one of these served as the validation set, while the others served as the inner training set. The model with the lowest normalized root-mean-square error was used as the reference, and the beta coefficients were applied to the outer test set to predict the ROCF-recall values. This process was repeated 4000 times, with subjects being randomly assigned to subgroups each time, to ensure that the results were not dependent on any specific division of the sample. Results underwent correction through false discovery rate (FDR) correction.^75^ The significance level was established at a *p*-value <0.05 after correction (pFDR).
