# Supplementary material for: Dynamic reconfiguration of aperiodic brain activity supports cognitive functioning in epilepsy: A neural fingerprint identification
Source: iScience. 2024 Nov 28;28(1):111497. doi: 10.1016/j.isci.2024.111497 (PMC11699349; doi:10.1016/j.isci.2024.111497)
Supplement: Document S1. Figures S1–S5 and Tables S1–S4 [file mmc1.pdf]

iScience, Volume *28*

Supplemental information

Dynamic reconfiguration of aperiodic brain activity supports cognitive functioning in epilepsy: A neural fingerprint identification

Emahnuel Troisi Lopez, Marie-Constance Corsi, Alberto Danieli, Lisa Antoniazzi, Marianna Angiolelli, Paolo Bonanni, Pierpaolo Sorrentino, and Gian Marco Duma

Supplemental information

	Iself		Iothers		Idiff		Iclinical	
	r	p	r	p	r	p	r	p
Number of Antiseizure Medications	0.17	0.254	-0.04	0.767	-0.09	0.546	-0.02	0.906

Table S1. Correlation between medications and fingerprint parameters, related to Figure 4. The table shows Pearson's correlation coefficients (r) and p-values. No significant ($p < 0.05$) correlation is present in the table.

	UTLE - Left	UTLE - Right	BTLE
Sample size	31	17	20
Age (years)	37.14 ± 16.1	43.88 ± 19.20	45.9 ± 16.01
Age of onset (years)	22.78 ± 16.11	28.51 ± 22.86	19.8 ± 13.93
Duration of Epilepsy (years)	14.41 ± 16.19	14.97 ± 17.36	26.7 ± 20.03
Number of Antiseizure Medications	1.58 ± 0.99	1.59 ± 0.71	2.75 ± 1.12

Table S2. Patients' subgroup details, related to STAR Methods. The table reports detailed information with respect to patients with unilateral temporal lobe epilepsy (UTLE) (UTLE - Left and UTLE - right, respectively), and patients with bilateral temporal lobe epilepsy (BTLE). Values are reported as mean ± standard deviation.

ID	Diagnosis	Age	Gender	Race	Ethnicity
SJ-1	UTLE-LEFT	47,03	M	White	White
SJ-4	UTLE-LEFT	48,62	F	White	White
SJ-5	UTLE-RIGHT	56,93	M	White	White
SJ-6	BTLE	57,55	M	White	White
SJ-7	UTLE-LEFT	60,82	M	White	White
SJ-8	UTLE-LEFT	42,57	F	White	White
SJ-9	BTLE	67,51	M	White	White
SJ-10	UTLE-LEFT	37,41	F	White	White
SJ-11	UTLE-RIGHT	33,64	M	White	White
SJ-12	UTLE-LEFT	20,41	M	White	White
SJ-13	BTLE	25,04	F	White	White
SJ-14	UTLE-LEFT	21,89	F	White	White
SJ-15	UTLE-LEFT	12,74	M	White	White
SJ-16	BTLE	23,79	M	White	White
SJ-17	UTLE-RIGHT	30,37	F	White	White
SJ-19	UTLE-LEFT	59,67	M	White	White
SJ-20	UTLE-RIGHT	51,94	M	White	White
SJ-21	UTLE-RIGHT	37,37	F	White	White
SJ-22	UTLE-LEFT	16,45	M	White	White
SJ-23	UTLE-RIGHT	75,06	M	White	White
SJ-24	UTLE-RIGHT	58,24	M	White	White
SJ-25	UTLE-LEFT	45,01	M	White	White
SJ-26	UTLE-LEFT	37,82	M	White	White
SJ-27	BTLE	42,56	F	White	White
SJ-28	UTLE-LEFT	32,21	F	White	White
SJ-29	BTLE	61,55	F	White	White
SJ-30	UTLE-LEFT	15,56	F	White	White
SJ-31	UTLE-LEFT	51,27	F	White	White
SJ-32	BTLE	45	M	White	White
SJ-33	UTLE-RIGHT	45	F	White	White
SJ-34	UTLE-RIGHT	75,54	F	White	White
SJ-35	UTLE-RIGHT	47,6	F	White	White
SJ-36	BTLE	62,33	F	White	White
SJ-37	UTLE-LEFT	41,31	M	White	White
SJ-38	UTLE-LEFT	30,54	F	White	White
SJ-39	UTLE-LEFT	17,37	M	White	White
SJ-40	UTLE-RIGHT	62,54	M	White	White
SJ-41	BTLE	49,57	F	White	White
SJ-42	UTLE-LEFT	16,52	M	White	White
SJ-43	UTLE-RIGHT	10,69	M	White	White
SJ-44	UTLE-LEFT	23,44	M	White	White
SJ-46	BTLE	31,87	F	White	White
SJ-47	UTLE-LEFT	43,07	F	White	White

SJ-48	UTLE-LEFT	64,84	M	White	White
SJ-49	UTLE-LEFT	37,76	F	White	White
SJ-50	UTLE-LEFT	20,54	M	White	White
SJ-51	BTLE	17,84	M	White	White
SJ-52	UTLE-LEFT	15,4	F	White	White
SJ-53	UTLE-LEFT	40,87	F	White	White
SJ-54	UTLE-RIGHT	15,52	F	White	White
SJ-55	UTLE-RIGHT	47,63	F	White	White
SJ-56	UTLE-RIGHT	24,55	M	White	White
SJ-57	UTLE-LEFT	40,21	F	White	White
SJ-58	UTLE-LEFT	9,21	M	White	White
SJ-59	UTLE-RIGHT	35,71	M	White	White
SJ-60	BTLE	64,48	M	White	White
SJ-61	UTLE-LEFT	56,88	M	White	White
SJ-62	BTLE	41,51	F	White	White
SJ-63	BTLE	51,48	F	White	White
SJ-64	BTLE	35,67	F	White	White
SJ-65	BTLE	31,32	F	White	White
SJ-66	BTLE	52,46	M	White	White
SJ-67	BTLE	74,09	M	White	White
SJ-68	BTLE	34,5	M	White	White
SJ-69	UTLE-LEFT	59,27	M	White	White
SJ-70	UTLE-LEFT	49,72	F	White	White
SJ-71	BTLE	51,94	F	White	White
SJ-72	UTLE-RIGHT	60,47	F	White	White

Table S3. Patients' details, related to STAR Methods. The table reports detailed individual information with respect to patients with unilateral temporal lobe epilepsy (UTLE) (UTLE - LEFT and UTLE - RIGHT, respectively), and patients with bilateral temporal lobe epilepsy (BTLE). Ancestry is not available

	SS			d.f.			MS		F	pFDR
	Groups	Error	Total	Groups	Error	Total	Groups	Error		
Iself	0.062	0.551	0.613	2	100	102	0.031	0.006	5.659	0.005
Iothers	0.29	0.184	0.474	2	100	102	0.145	0.002	78.891	< 0.001
Idiff	0.617	0.778	1.395	2	100	102	0.308	0.008	39.628	< 0.001

Table S4. Statistical replication analysis, related to STAR Methods. The table reports statistics data of the omnibus test performed using PERMANOVA (10,000 iterations) on fingerprint parameters (Iself, Iothers, Idiff) calculated on 180 seconds of recording (the maximum available time equally available across all participants). SS = sum of squares; d.f. = degrees of freedom; MS = mean square. pFDR = p-value after false discovery rate correction.

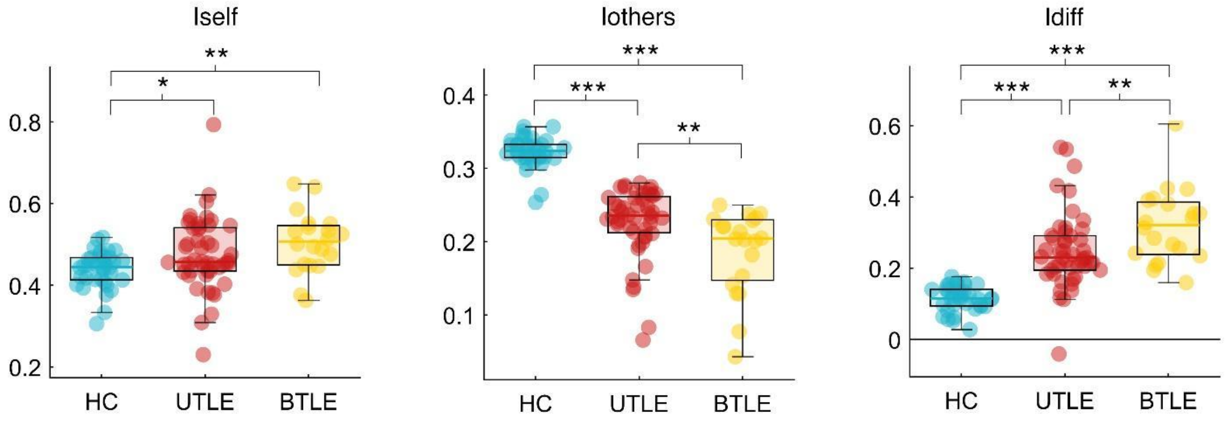

Figure S1. Fingerprinting analysis with full (180 seconds) test-retest recordings, related to Figure 2. Statistical comparison of fingerprint parameters (i.e., Iself, Iothers, and Idiff) between healthy controls (HC; n = 35), patients with unilateral temporal lobe epilepsy (both left and right) (UTLE; n = 48) and patients with bilateral temporal lobe epilepsy (BTLE; n = 20). The box plot includes data from 25 to 75th percentiles; the median is represented by the horizontal line inside each box; error lines reach the 10th and 90th percentiles; filled circles represent the observations. Omnibus test: PERMANOVA; post-hoc analysis: permutation test; number of permutations: 10'000; p-values were corrected through false discovery rate (pFDR), and significance was assessed as follows: * < 0.05, ** < 0.01, *** < 0.001.

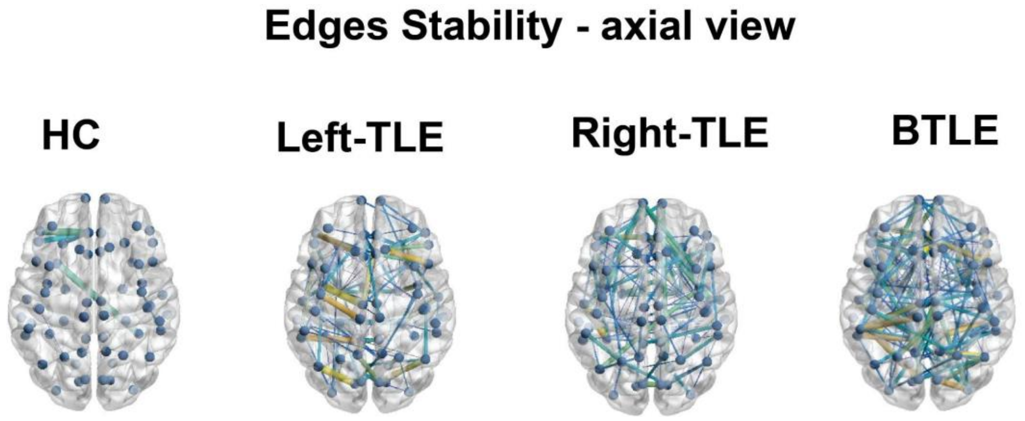

Figure S2. Edges' stability, top view, related to Figure 3. The figure shows the edge-wise stability from the top view; dots represent the brain regions; bars connecting the dots represent the stability of the link between two given nodes. The higher the stability, the thicker the link. HC: healthy controls; Left-TLE: patients with left temporal lobe epilepsy; Right-TLE: patients with right temporal lobe epilepsy; BTLE: patients with bilateral temporal lobe epilepsy

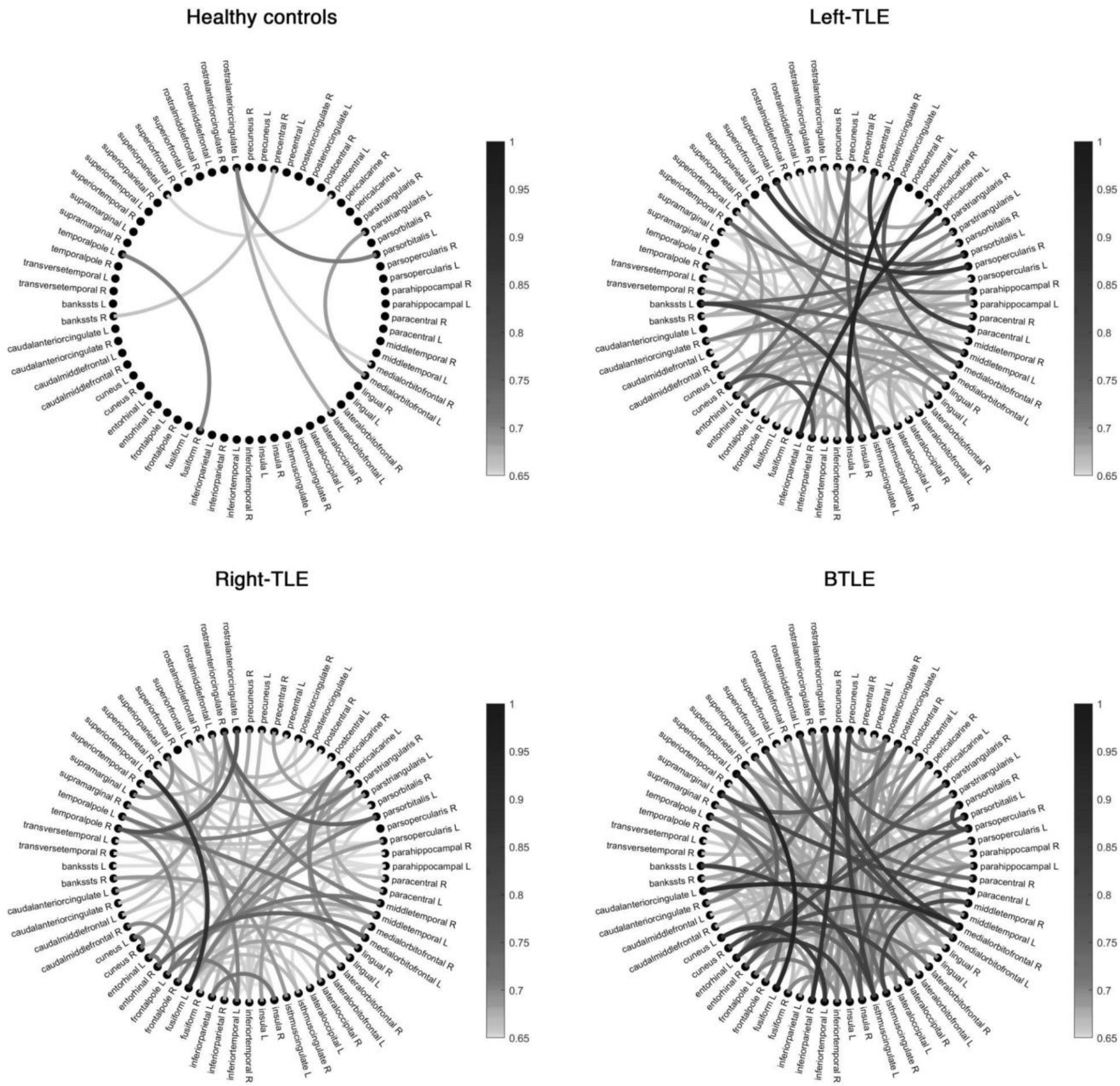

Figure S3. Circular plot of edges' stability, related to Figure 3. These plots allow to individuate the regions whose links display high stability. For visualization purposes, only edges with ICC ≥ 0.65 are displayed. Left-TLE: patients with left epilepsy; Right-TLE: patients with right temporal epilepsy; BTLE: patients with bilateral temporal lobe epilepsy.

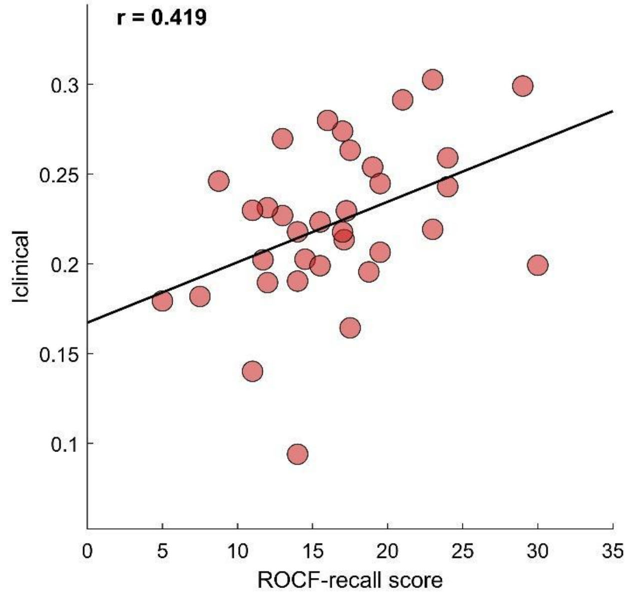

Figure S4. Clinical correlation with full recordings (180 seconds), related to Figure 4. The figure shows the scatter plot of the correlation between Iclinical values (i.e., similarity of the pattern of brain dynamics between a patient with the average of the control group) and recalling performance scores during the Rey–Osterrieth complex figure test (ROCF-recall), with regards to the patients with unitemporal epilepsy. Red dots represent the observations. Statistical test: Pearson correlation test ($r = 0.481$, $p = 0.014$); $n = 34$ out of 48 total patients with unitemporal epilepsy, as clinical data were not available for 14 patients.

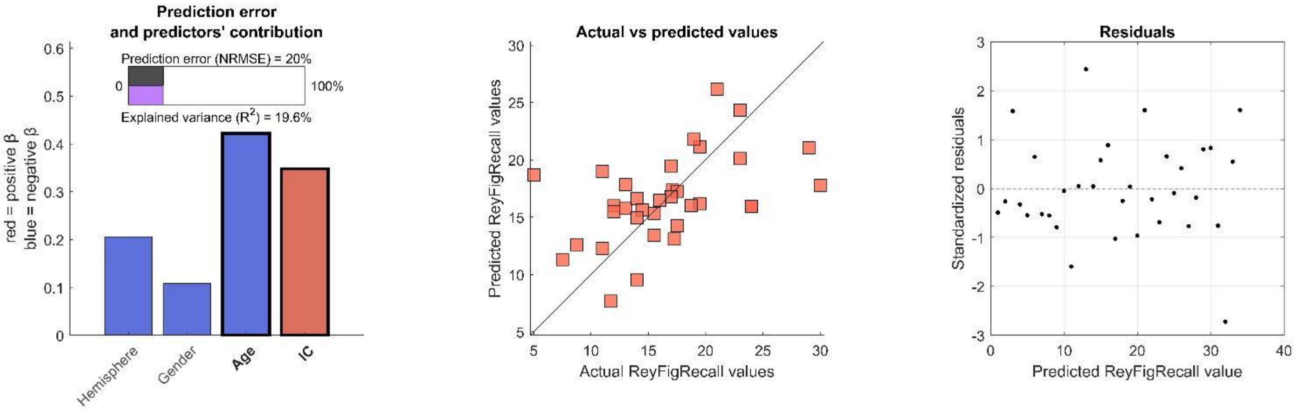

Figure S5. Multilinear regression model for clinical prediction based on maximum available recording duration (180 seconds), related to Figure 5. The figure shows the results of the multilinear model with nested 5-fold cross-validation. The multilinear model significantly predicts the scores of the Rey–Osterrieth complex figure recall test (ROCF-recall) in patients with unilateral epilepsy ($F(4,29) = 4.39$, $p = 0.008$)). The model is based on four predictors (i.e., lateralization of the condition (Hemisphere, in figure), Gender, Age, and Iclinical (IC)). The left panel report the statistics of the model; predictors' values are z-scored in order to make the beta coefficients comparable; significant predictors are reported in bold (Age, $\beta = -0.42$, p

= 0.019; IC, $\beta = 0.35$, $p = 0.048$); NRMSE: normalized root mean square error is equal to 20%; explained variance is represented by R2 measure, equal to 19.6%. The middle panel is a scatter plot that compares the actual ROCF-recall scores with the ROCF-recall scores predicted by the model. The more the predictors are aligned along the diagonal, the higher is the accuracy of the prediction; Spearman correlation coefficient = 0.671. Finally, the third panel shows the distribution of the standardized residuals. Sample size is n = 34, out of 48 total patients with unitemporal epilepsy, as clinical data were not available for 14 patients.
